# Focus on the agents most frequently responsible for perioperative anaphylaxis

**DOI:** 10.1186/s12948-018-0094-7

**Published:** 2018-07-09

**Authors:** E. Di Leo, P. Delle Donne, G. F. Calogiuri, L. Macchia, E. Nettis

**Affiliations:** 1 Unit of Internal Medicine, Section of Allergy and Clinical Immunology, “F. Miulli” Hospital, Acquaviva delle Fonti, BA Italy; 2 Department of Emergency and Organ Transplantation, School and Chair of Allergology and Clinical Immunology, University of Bari—Aldo Moro, Bari, Italy; 3 Pneumology Department, Sacro Cuore Hospital, Gallipoli, Lecce, Italy; 4 Policlinico, Piazza Giulio Cesare, 11, 70124 Bari, Italy

**Keywords:** Perioperative allergy, Latex, Anaphylaxis, Neuromuscular blocking agents

## Abstract

Adverse reactions (ARs) to drugs administered during general anesthesia may be very severe and life-threatening, with a mortality rate ranging from 3 to 9%. The adverse reactions to drugs may be IgE and non-IgE-mediated. Neuromuscular blocking agents (NMBA) represent the first cause of perioperative reactions during general anesthesia followed by latex, antibiotics, hypnotic agents, opioids, colloids, dyes and antiseptics (chlorhexidine). All these substances (i.e. NMBA, anesthetics, antibiotics, latex devices) may cause severe systemic non-IgE-mediated reactions or fatal anaphylactic events even in the absence of any evident risk factor in the patient’s anamnesis. For this reason, in order to minimize perioperative anaphylactic reactions, it is important to have rapid, specific, sensitive in vitro diagnostic tests able to confirm the clinical diagnosis of acute anaphylaxis.

## Background

The potential occurrence of hypersensitivity reactions (IgE-mediated or non-IgE-mediated) is a great concern for anesthesiologists, because adverse reactions (ARs) may be very severe and life-threatening, with a mortality rate ranging from 3 to 9% [1]. Fatal anaphylaxis as well as a great proportion of less severe anaphylactic reactions is caused by drugs and substances related to general anesthesia [2, 3].

The individuation of the culprit agent is very difficult because of the several drugs administered during general anesthesia. Neuromuscular blocking agents (NMBA), latex, antibiotics, induction agents and opiates are the most common substances incriminated in perioperative anaphylaxis [4–6]. Perioperative reactions depend on the underlying mechanism classified in 2 groups: reactions resulting from direct nonspecific mast cell and basophil activation (severe systemic non-IgE-mediated hypersensitivity reactions) and IgE-dependent allergic reactions (IgE-mediated, anaphylaxis) [7].

## Incidence

The incidence of anaphylaxis is estimated between 1 in 10,000 and 1 in 20,000 anesthetic procedures [2, 3]. However, anaphylactic reactions cannot be clinically distinguished from non-immune mediated reactions which account for 30–40% of hypersensitivity reactions [1, 8].

An IgE-mediated mechanism has been confirmed in 40–70% of cases [9]. Generally reactions resulting from direct histamine release are usually less severe than IgE-mediated reactions [10]. In a recent study on anaphylaxis during general anesthesia, an IgE-mediated cause was identified in 103 patients (64%); NMBA constituted the leading cause (38%) followed by antibiotics (8%), patent blue dye (6%), chlorhexidine (5%) and other agents (7%) [11].

In recent decades perioperative adverse reactions had more attention and the general view is that immediate-type hypersensitivity reactions are largely under reported and causes remain often unknown. In addition, the extremely variable rate of reactions is due to the lack of homogeneity of clinical criteria and diagnostic tests, and the diversity of the populations studied [12].

In case of IgE-mediated perioperative anaphylaxis, NMBA represent the first cause whereas latex is the second responsible allergen, followed by antibiotics [12–15]. About 20% of perioperative anaphylaxis is attributed to latex [16] even if the prevalence of anaphylaxis to latex been declining in the last years due to a highly efficient prevention strategy [11, 17]. Other agents used during general anesthesia are also implied as anaphylaxis causatives or involved in anaphylactic response, such as hypnotic agents, opioids, colloids, dyes, and antiseptics (chlorhexidine) [13]. Non steroidal anti-inflammatory drugs (NSAIDs) and iodinated contrast agents may provoke non allergic anaphylaxis, although also for these drugs IgE-mediated reactions are hypothesized [13].

## Neuromuscular blocking agents

NMBA (muscle relaxants) are responsible for 60–70% of anaphylactic episodes during general anesthesia or in the postoperative setting [9, 12], thus representing the most common cause of perioperative reactions in France [8] with an 1:6500 incidence [10]. The NMBAs include: succinylcholine, benzylisoquinolines (atracurium, cisatracurium, doxacurium, mivacurium) and aminosteroids or benzylisoquinolinium-type NMBA: pancuronium, rapacuronium, rocuronium, vecuronium. Hypersensitivity reactions to NMBA may be either IgE or not-IgE mediated in their pathogenesis. An IgE-mediated response is due to the quaternary ammonium (NH4+) structures that represents the main antigenic epitope of NMBAs [18], while the second one is provoked by benzylisoquinolinium-type NMBA (aminosteroids) such as mivacurium, atracurium, and d-tubocurarine that are responsible for non-IgE-mediated hypersensitivity reactions [19]. From 20 to 50% of adverse reactions to NMBAs are considered to result from direct nonspecific mast cell and basophil activation [10]. Histamine release is predominantly found with the use of the d-tubocurarine, atracurium, and mivacurium among the benzylisoquinolines, and rapacuronium among the aminosteroids [20].

Unfortunately, quaternary ammonium groups are ubiquitous epitopes, which are contained in other drugs or disinfectants, so such reactive groups are responsible for cross-reactivity phenomenon with other drugs.

The presence in NMBA of ubiquitous epitopes such as a substituted ammonium group (that are likely the immunodominant determinant recognized by IgE) is responsible for high cross-reactivity among these drugs (most consistently between pancuronium, rocuronium and vecuronium) [21], but cross-reactions also may occur between muscle relaxants and other classes of pharmaceuticals: acetylcholine, choline, morphine, neostigmine, pentolinium and pholcodine [22].

Pholcodine, for instance, is a potentially sensitizing antitussive agent with similar structure to morphine and NMBAs, which was able to induce the production of IgE antibodies cross-reacting with suxamethonium [23]. In addition, Fisher was the first to hypothesize the presence of specific IgE versus natural products containing quaternary ammonium such as foods, cosmetics (tioglycol ammonium), eyedrop preservatives (benzalconium chloride), disinfectants, and industrial materials [24].

In the light of these findings, previous exposure to non-anesthetic drugs or other substances may induce a hidden sensitization to muscle-relaxing agents, resulting in reactions among patients without prior anesthesia.

The commonest triggers of NMBA reactions were found to be the suxamethonium (the most involved), followed by rocuronium, and then in descending order by vecuronium, pancuronium and atracurium [21]. Several studies have been conducted to identify possible risk factors for adverse reactions to muscle relaxants, and to evaluate the predictive value of skin tests with muscle relaxants with discordant conclusions. At present, however, an allergic work upon the implementation of an investigative systematic preoperative screening in the general population for the potential of anaphylaxis from NMBA is not recommended [10].

## Natural rubber latex

Latex (*Hevea brasiliensis*) is a long-lasting, resistant and elastic product, which allows to maintain good tactile sensitivity and it protects against diseases transmitted through body fluids, so latex-derived products are largely present in hospital setting (e.g., gloves, catheters, bottles with pierceable septum, tourniquets, AMBU bags, anesthesiological masks, cannulas, drainages, endotracheal tubes, bandages) [7, 25]. Latex represents an important cause of anaphylaxis during anesthesia [26] because it is the second cause of adverse reactions during perioperative period [7, 25, 27]. Multiple surgery procedures in spina bifida patients and professional exposure are associated with an increased frequency of latex allergy. Identification of patients belonging to at risk population, patients already sensitized to latex and medical setting latex-free use, have already allowed to reduce the incidence of perioperative latex adverse reactions. Exposure to latex may occur by contact through skin and mucosae or by inhalation of airborne particles [28]. The risk of developing an allergy to latex is greater for some groups: healthcare workers, children requiring multiple or repetitive surgical and medical procedures (e.g., spina bifida, spinal cord trauma, and urogenital malformations) that need chronic bladder care with repeated insertion of natural rubber latex (NRL) catheters or chronic indwelling catheters [29], housewives or workers occupationally exposed (hairdressers, gardeners, workers in the rubber industry) [30]. Obstetric population appears more prone to develop sensitizations to NRL proteins probably due to repeated contact between latex gloves and mucosal membranes during vaginal examinations or delivery [31].

As latex allergy is suspected by clinical history (symptoms like urticaria or angioedema when skin or mucosa come into contact with manufacts such as balloon, gloves, diving masks, condoms, etc. or during medical examination by latex medical devices), patients have to undergo skin prick test (SPT) and serum-specific IgE tests. SPTs are usually performed with several commercial extracts and with extemporary preparations at various dilutions obtained from high allergens content latex gloves and represents the gold standard in the diagnosis of latex allergy, characterized by high sensitivity and specificity [32].

Serum-specific IgE can be detected through molecular diagnosis by ImmunoCAP/ISAC method (Thermofisher, Uppsala, Sweden) that is able to distinguish between a true sensitization and an asymptomatic cross-reactivity. Latex allergens Hev b 1, Hev b 3, Hev b 5 and Hev b 6 are currently considered markers of genuine latex sensitization [33, 34]. On the other hand, latex-sensitized subjects with a positive serum specific IgE against latex, but negative SPT and without latex-specific symptoms upon contact with latex-containing material, have a profilin sensitization with monosensitization to Hev b 8 [34]. These patients can undergo major surgery in normal surgical setting without any consequences since Hev b 8 represents a marker of asymptomatic latex sensitization [34]. Finally, if latex allergy is strongly suspected and IgE tests gave negative results, a challenge test should be performed by wearing a latex glove to the patient for increasing periods, monitoring for objective signs of an allergic reaction [28].

In sensitized patients, the exposure to NRL proteins can provoke a type I IgE-mediated hypersensitivity reaction and a type IV reaction (not life threatening) responsible for a contact dermatitis that elicits slow-onset eczematous reactions. IgE-mediated reactions may involve various organs and systems, even simultaneously. Clinical manifestations, that occur within a few minutes after contact with NRL proteins, include urticaria, angioedema, conjunctivitis, allergic rhinitis, asthma and anaphylaxis. Immediate reactions, such as anaphylaxis, can be life threatening [28, 35] and may occur after a direct contact with latex, usually gloves, or instruments, or with aerosolization of latex antigen adherent to the cornstarch powder of latex gloves when gloves are donned during operative procedures. The administration of drug through a latex port prior to surgery may cause an intraoperative latex anaphylaxis that is usually also related to the surgical procedure itself [36].

Airborne exposure to latex may also cause a contact dermatitis. It is a non-immune-mediate reaction that appears at the side of contact and cause itching, redness, blisters [35].

Primary prevention is the avoidance of sensitization in at-risk population whereas secondary prevention means avoiding the onset of the allergic reaction in previously sensitized patients by establishing a “latex-free environment” not only in operating rooms, but also in all recovery rooms [37–39]. Several studies performed in different countries demonstrated that using powder-free latex gloves and replacing NRL with another material, make it possible to decrease perioperative latex allergy incidence [40]. Recent findings demonstrate the effectiveness of sublingual immunotherapy in patients with history of latex allergy inducing an effective desensitization to NRL [38, 41].

## Antibiotics

Penicillins and cephalosporins are responsible for 70% of perioperative anaphylactic reactions induced by antibiotics [42]. The reactions induced by individual antibiotics are mostly observed after penicillins (0.004–0.015%) and cephalosporins (0.0001–0.1%) [43]. Their frequency has increased over the last 20 years [24]. Currently, allergy to b-lactams represents 12–15% of the perioperative reactions observed in France [44]. The incidence of reactions provoked by these drugs has been increasing due to a widespread use of them for the perioperative antibiotic prophylaxis [45], but also due to the worldwide use of them in the general population. The prevalence of penicillin allergy in the general population varies between 0.7 and 10% [10].

Betalactam antibiotics, vancomycin and quinolones are frequently implicated in adverse reactions during perioperative period representing the third cause of anaphylactic reactions in the surgical patients [46].

Heterogeneous and various mechanisms are involved in adverse drug reactions due to penicillins and other betalactams such as cephalosporins both IgE-mediated and non IgE-mediated in addition to unknown mechanisms.

According to EAACI interest group on drug hypersensitivity, the diagnostic protocol for allergic reactions to b-lactam [47] contemplate dosage of serum IgE for several penicillin determinants [Thermofisher, Sweden, penicilloyl G (c1), penicilloyl V (c2), amoxycilloyl (c6), ampicilloyl (c5) and cefaclor (c7)]. Because beta-lactams specific IgE dosage is generally less sensitive than skin tests, that they represent valuable and safe tools in the diagnostic approach for patients with suspected IgE-mediated beta-lactam allergy [13, 48–50] the investigation methods include in vivo tests, such as SPT and IDT. For SPT the suspected agent can be tested, in addition to the available commercial reagents, such as benzylpenicilloylpoly-l-lysine (PPL) and so-called minor determinants mixture (MDM), including amoxicillin (AX) and ampicillin (AMP) [12, 13].

Provocation tests with b-lactam antibiotics should be performed only in patients with a positive clinical history but negative IgE and skin test investigations [47].

Vancomycin, a glycopeptide antibiotic usually used for treatment of Gram positive resistant organisms and for patients with penicillin allergy, may provoke reactions both IgE-mediated and non IgE-mediated. Typically Vancomycin is responsible for “red man syndrome” consisting in non-IgE-mediated hypersensitivity reactions with flushing, pruritus, an erythematous rash of the head and upper torso, and arterial hypotension. These non-immunologic reactions are associated with a rapid infusion of the drug at the first dose [12, 51]. Sometimes dyspnea, angioedema, and hypotension can occur [12].

Quinolones represent the third most important group of antibiotics involved in perioperative anaphylaxis [46]. They can induce hypersensitivity reactions mediated by IgE and T cells, in addition to non IgE-mediated reactions. IgE-mediated reactions are more common and are severe in over 70% of cases [10]; the most frequent clinical manifestations are anaphylaxis and anaphylactic shock [10]. Diagnostic methods (skin tests or sIgE assays) for quinolones hypersensitivity reactions are not validated, even if in about 50% of patients with quinolones anaphylaxis IgE antibodies were found [52]. Other antibiotics that are often used in the operating room and that may rarely trigger an anaphylactic reaction include clindamycin, gentamicin, and metronidazole. Really, every antibiotic represents a potential cause of anaphylaxis [43].

## General anesthetics

Among the barbiturate anesthetics, in the past years, thiopental was in the most frequent agent responsible for anaphylaxis during anesthesia, but in more recent surveys, anaphylaxis from thiopental sodium is only rarely described because of the its less intensive application [12].

Generally, although their frequent application, anaphylaxis from non-barbiturate hypnotics is rare [12].

Propofol is a hypnotic agent that has been associated with anaphylactic reactions [13]. Despite the propofol is formulated in a lipid solution containing 10% soybean oil, 2.25% glycerol, and 1.2% egg lecithin [13], the presence of these components is not related with an increased risk of anaphylactic reactions due to propofol in patients with egg or soy allergy [13]. Immediate reactions involving propofol are most frequently due to an alkyl phenol that bears two isopropyl groups that may act as antigenic epitopes.

Propofol allergy has been diagnosed by skin tests, by sIgE and by histamine-release tests, although propofol can induce also a direct release of histamine concentration-dependent [53].

Yet, benzodiazepines are rarely a cause of immediate hypersensitivity reactions. Among this drug class, midazolam is the main causative agent in the perioperative setting [12, 46], and SPT with the undiluted drug and intradermal test can be performed to investigate about the reaction [46].

## Conclusions

In the perioperative period patients are exposed to many substances (i.e., anesthetics, antibiotics, latex devices, blood preparations, heparin, fluids) to ensure maximum safety and best care. All these substances may cause severe systemic non-IgE-mediated reactions or fatal anaphylactic events even in the absence of any evident risk factor in the patient’s anamnesis. It is important to keep in mind that is not always possible to detect certainly the culprit agent of a perioperative anaphylaxis because the cause-effect relationship is often difficult to demonstrate. In addition the diagnosis is not easy to make in an anesthetized patient because there are technical objective difficulties to recognize a hypersensitivity reaction in a perioperative setting. Firstly, the patient is unconscious and uncompliant, so anesthetists may only control vital signs and sudden changes in cardiac rate, a drop in blood pressure or a desaturation in pulse-oxymetry is not sufficient to suspect the onset of an immediate type hypersensitivity because such modifications may be associated with other pathologic conditions, i.e., a spontaneous pneumothorax, myocardial infarction or severe arrhythmia, which an anesthetists must consider rapidly.

Muscle relaxants, NRL and antibiotics are the most common anesthetic drugs or substances that may be involved in anaphylaxis.

To prevent successive anaphylaxis events, it is important to document every ARs and, above all, substances and/or drugs used during anesthesia, so that the identification of the causative drug by allergist can be simpler. Once the diagnosis has been made, the patient must be informed about symptoms during the anesthesia, the drugs that have been responsible for the reaction and must be provided with a bracelet or a tag that shows in detail the drugs to which he is allergic.

Prevention is an important component to decrease the incidence of anaphylaxis especially for latex which is the second cause of anaphylactic reactions in perioperative period. Often this is underestimated because when ARs occur, the drugs are considered mainly responsible [30]. Perioperative allergy to latex can be limited if an accurate anamnesis is made about previous adverse reactions to NRL manufacts and latex-related food, so it is possible to suspect sensitization to latex and to start diagnostic protocols. Avoiding latex exposition in medical settings is the only way to decrease new sensitizations and to reduce incidence of ARs.

In realty, the greatest necessity, in order to minimize perioperative anaphylactic reactions, is to have rapid, specific, sensitive in vitro diagnostic tests able to confirm the clinical diagnosis of acute anaphylaxis.
